# Acyclovir-Loaded Chitosan Nanospheres from Nano-Emulsion Templating for the Topical Treatment of Herpesviruses Infections

**DOI:** 10.3390/pharmaceutics10020046

**Published:** 2018-04-10

**Authors:** Manuela Donalisio, Federica Leone, Andrea Civra, Rita Spagnolo, Ozgen Ozer, David Lembo, Roberta Cavalli

**Affiliations:** 1 Department of Clinical and Biological Sciences, University of Turin, San Luigi Gonzaga Hospital, Regione Gonzole 10, Orbassano, 10043 Turin, Italy; manuela.donalisio@unito.it (M.D.); andrea.civra@unito.it (A.C.); david.lembo@unito.it (D.L.); 2 Department of Drug Science and Technology, University of Turin, via Pietro Giuria 9, 10125 Turin, Italy; federica.leone@polito.it (F.L.); rita.spagnolo@unito.it (R.S.); 3 Department of Applied Science and Technology, Politecnico of Turin, Corso Duca degli Abruzzi 24, 10129 Turin, Italy; 4 Faculty of Pharmacy, Department of Pharmaceutical Technology, EGE University, 35100 Izmir, Turkey; ozgen.ozer@ege.edu.tr

**Keywords:** acyclovir, chitosan nanospheres, antiviral activity, topical infections

## Abstract

Acyclovir is not a good candidate for passive permeation since its polarity and solubility limit is partitioning into the stratum corneum. This work aims to develop a new topical formulation for the acyclovir delivery. New chitosan nanospheres (NS) were prepared by a modified nano-emulsion template method. Chitosan NS were characterized by Dynamic Light Scattering (DLS), Transmission Electron Microscopy (TEM), and an in vitro release study. The in vitro skin permeation experiment was carried out using Franz cells and was equipped with porcine skin. Biological studies were performed on the Vero cell line infected by HSV-1 and HSV-2 strains. The acyclovir loaded chitosan NS appeared with a spherical shape, a size of about 200 nm, and a negative zeta potential of about 40.0 mV. The loading capacity of the drug was about 8.5%. In vitro release demonstrated that the percentage of acyclovir delivered from the nanospheres was approximately 30% after six hours. The in vitro skin permeation studies confirmed an improved amount of permeated acyclovir. The acyclovir-NS complex displayed a higher antiviral activity than that of free acyclovir against both the HSV-1 and the HSV-2 strain. The acyclovir-loaded NS showed no anti-proliferative activity and no signs of cytotoxicity induced by NS was detected. Confocal laser scanning microscopy confirmed that the NS are taken up by the cells.

## 1. Introduction

Herpesviruses are responsible for chronic, life-long viral infections that range from asymptomatic infections or mild cutaneous and mucocutaneous lesions up to severe clinical manifestations as encephalitis and aseptic meningitis mainly in immunocompromised individuals and newborns [1]. After the primary infection of the skin or mucosa, viruses establish latency into root ganglia and may intermittently reactivate causing recurrent episodes with or without clinical signs. Herpes Simplex Virus Type 1 (HSV-1) is a highly contagious virus and is primarily transmitted by oral–oral contact, which is common and endemic throughout the world. An estimated 3.7 billion people under the age of 50 have had an HSV-1 infection [2]. HSV-1 primary infections generally occur during childhood as asymptomatic infections or as herpetic gingivostomatitis conditions while recurrent manifestations appear as orolabial lesions, which are called cold sores, fever blisters, or generally herpes labialis. On the other hand, HSV-2, which is mainly sexually transmitted, is responsible for genital herpes, which affected an estimated 417 million people worldwide in 2012 [1,2]. Furthermore, the HSV-2 infection increases the risk of acquiring an HIV infection by approximately three-fold and genital herpes can occur in 60–90% of HIV-infected people [3]. In developed countries, initial genital herpes episodes caused by HSV-1 are increasing especially among young people [4].

Different anti-herpetic drugs such as acyclovir, famciclovir, and valacyclovir are approved and used to treat acute symptomatic herpetic infections. These drugs reduce the severity and frequency of symptoms, but they cannot cure and eradicate the latent infections. Recommendations for the treatment of genital herpes suggest the use of systemic antiviral drugs, which are orally administered, to control the signs and symptoms of first clinical and recurrent genital episodes [5]. On the other hand, treatment of recurrent orolabial mucocutaneous herpes is commonly based on topical antiviral agents in order to speed up healing of the sores and decrease symptoms such as tingling, pain, burning, and itching [6]. Acyclovir (9-[(2-hydroxyethoxy)methyl])-9H-guanine), which is a synthetic nucleoside analogue derived from guanosine, is the drug of choice for treating HSV infections. However, due to its short half-life and incomplete absorption, it must be taken repeatedly throughout the day to be effective. Its oral dosage form requires five administrations daily (up to 1200 mg/day) and the dosage interval for intravenous formulations is eight hours [7]. To overcome this limitation some derivatives of acyclovir have been developed. For example, valacyclovir, the L-valyl ester of acyclovir, which displays higher oral bioavailability (30–50%), requires gram doses to be administered orally (1000 mg three times a day) in order to treat a local infection [8].

In local therapy, acyclovir is not a good candidate for passive permeation since its polarity (log D = −1.76 at pH 7) and its sparingly solubility in both aqueous and lipid media limit its partitioning into the *stratum corneum*. For these reasons, a high frequency of topical application is required. The cream should be applied about five times a day (every three to four hours) for 4 consecutive days for cold sores [6].

In this context, an efficient topical acyclovir delivery is highly desirable because it enables targeted therapy, reduces circulating drug levels, and attenuates the risk of renal insufficiency. Most clinical studies have shown that the current topical formulations of acyclovir used against herpes labialis are unable to reach the basal epidermis target site where the virus is found, which results in delayed antiviral activity and sub-inhibitory concentrations in the basal epidermis, which translate into poor clinical efficacy [9]. The present work aims to develop a successful new topical formulation for the delivery of acyclovir that is able to increase the efficacy of the currently available commercial products for the topical treatment of Herpes simplex virus infections. Additionally, topical administration is the favored route for local delivery of therapeutic agents due to its convenience, patient compliance, and affordability. The specific challenge of designing a therapeutic system is to achieve an optimal local concentration of a certain drug for an appropriate duration of time. In the last decade, more research focused its attention on topical drug delivery approaches that exploit nanoscale systems to obtain a sustained release and maintain a localized prolonged therapeutic effect [10]. The nanotechnology-based strategy might be challenging for the treatment of skin problems that are difficult to address such as infectious diseases.

The use of nanodelivery systems, either lipophilic or hydrophilic, to improve the percutaneous absorption of acyclovir has been previously described. Solid Lipid Nanoparticles (SLN) tuned to incorporate acyclovir improved the dermal delivery in the rat skin model, which suggests their use for the treatment of Herpes Simplex topical infections [11]. Carboxylated nanosponges have been designed to improve the acyclovir loading in the nano-carrier and the drug’s effectiveness. A remarkable in vitro antiviral efficacy was observed when acyclovir was encapsulated within the nanocarrier structure [12]. Al-Subaie et al. designed a nano-emulsion containing a permeation enhancer and dispersed it in a chitosan gel for topical application. This formulation showed a two-fold higher drug permeation than the control in ex-vivo studies [13].

The goal of this work was to prepare chitosan nanospheres (NS) by a purposely modified nano-emulsion templating method [14] to increase the local acyclovir concentration and favor the local drug accumulation. The use of natural materials was taken into consideration due to the enormous interest they have gained as biomaterials for human healthcare systems [15]. Chitosan, a natural linear polycationic polysaccharide, consists of glucosamine and N-acetylglucosamine monomers linked through β (1-4) glycosidic linkages and is structurally similar to glycosaminoglycan (GAG) and its analogs. Due to its biocompatibility, low toxicity, and biodegradability, chitosan is considered a Generally Recognized as Safe (GRAS) component and its chloride salt is reported in a monograph of the European Pharmacopoeia. It is of significant interest for industrial and biomedical applications because it is a cheap, abundant waste product of the food industry with excellent biocompatibility, degradability, and unique structural and physicochemical characteristics. Based on these premises, chitosan is an interesting biomaterial for a number of pharmaceutical and cosmetic applications [16]. Furthermore, chitosan is involved in wound healing and regeneration processes [17,18]. Moreover, acyclovir permeation through porcine skin after topical application of loaded NS dispersed in a gel was determined using static Franz cells. Biological assays were carried out to assess the in vitro antiviral activity of acyclovir-loaded NS against HSV-1 and HSV-2 and their effect on cell viability.

## 2. Materials and Methods

### 2.1. Materials

Acyclovir, chitosan (medium MW, deacetylation 75–85%), fluoresceine isothiocianate, Tween 80, and Arlacel 83 were purchased from Sigma (Milano, Italy). Solvents and reagents, unless otherwise indicated, were analytical-grade commercial products and used as received.

### 2.2. Preparation of Acyclovir-Loaded Chitosan Nanospheres

The preparation method exploits a nano-emulsion as a template in order to obtain nano-sized particles starting from nano-sized emulsion droplets. A purposely-tuned W/O nano-emulsion was prepared and then the reduction of the droplet size was obtained by sonication (Sonicator Ultrasonic Processor XL2020, Misonix Incorporated, 1938, New Highway, Farmingdale, NY, USA), frequency 20 MHz). The nano-emulsion components were FDA-accepted excipients. The external phase consisted of a mixture of mineral oil and dodecanol (1:1, *v*/*v*) while the internal phase was a chitosan acetic acid solution (1% *w*/*w*). Tween 80 (0.50%) and Arlacel 83 (3.25%) were used as surfactants. To obtain the nanospheres, the nano-emulsion was dropped by a syringe using a roller pump in a sodium citrate aqueous solution (0.1 M) under stirring. This semi-automatic technique ensures more reproducibility for the preparation process, which was previously reported [19]. After completing the drift phase, the system was centrifuged and washed three times with water. Finally, the aqueous suspension of nanoparticles was filtered.

In order to incorporate the drug in the nanospheres, a series of preliminary experiments were carried out. These experiments aimed at optimizing the drug amount and the stability of the system. The tuned acyclovir-loaded chitosan nanospheres were obtained with the same procedure in the presence of the drug (10 mg/mL) in the internal aqueous phase.

A volume of NS aqueous nano-suspension was freeze-dried using a Modulyo Freeze-dryer (Edwards, Crawley, UK)) to obtain a solid powder.

### 2.3. Preparation of Fluorescent-Labelled Chitosan Nanospheres

Fluorescent chitosan NS were obtained by labeling freeze-dried pre-formed chitosan NS with fluorescein isothiocyanate (FITC), which was previously reported. For this purpose, an FITC dimethylsulfoxide solution (10 mg/mL) was added to the chitosan nanosphere suspension under stirring for 24 h at room temperature in the dark. At the end of the labeling procedure, an aliquote of NS aqueous nano-suspension was freeze-dried using a Modulyo Freeze-dryer (Edwards, Crawley, UK) to obtain a solid powder.

### 2.4. Characterization of Acyclovir-Loaded Nanospheres

Average particle diameter, polydispersity index, and Z potential of the acyclovir-loaded NS, fluorescent-labelled NS, and blank NS were determined by Photon Correlation Spectroscopy using a 90 Plus particle sizer (Brookhaven Instruments Corporation, Holtsville, NY, USA), equipped with MAS OPTION particle sizing software (Brookhaven Instruments Corporation). The measurements were made at a fixed angle of 90° for all samples, which were suitably diluted with filtered distilled water for every measurement. Zeta potential measurements were also made using an additional electrode in the same instrument. For zeta potential determination, samples of the three formulations were diluted with 0.1 mM KCl and placed in the electrophoretic cell in which an electric field of about 15 V/cm was applied.

### 2.5. Morphology Determination

The morphology of the acyclovir-loaded NS was observed by Trasmission Electron Microscopy (TEM). Transmission electron microscopy (TEM) was employed to evaluate the particle shape and morphology. Philips CM 10 transmission electron microscope (Philips Electron Optics, Eindhoven, the Netherlands) was used and the particle size was measured using the NIH image software (NIH, Bethesda, MD, USA). The nanosponge suspensions were sprayed on Formwar-coated copper grid and air-dried before observation.

### 2.6. Quantitative Determination of Acyclovir

The quantitative determination of acyclovir was carried out by HPLC analysis using a Perkin Elmer instrument (L2 Binary Pump, Perkin Elmer, Waltham, MA, USA) with a UV-vis spectrophotometer detector (LC 95, Perkin Elmer). A reverse-phase hypersil octadecyl-silica (ODS) column (25 cm × 4.6 mm Varian, Agilent Technologies, Santa Clara, CA, USA) was used with a mobile phase consisting of a 12:88 (*v*/*v*) ratio of acetonitrile: 20 mM ammonium acetate buffer pH = 3.5 and a flow rate of 1 mL/min. The UV detector wavelength was set to 250 nm. The acyclovir concentration was calculated using an external standard method from a standard calibration curve. The acyclovir calibration curve was linear in the range 0.5–15 μg/mL with r^2^ of 0.9997.

### 2.7. Determination of Acyclovir Encapsulation Efficiency and Loading Capacity

The loading capacity was determined from freeze-dried loaded samples. A weighted amount of freeze-dried acyclovir-loaded NS (about 3 mg) was dispersed in 5 mL of distilled water at a pH equal to 2.3 and adjusted by HCl. After sonication for 15 min and centrifugation at 5000 rpm for 20 min, the supernatant was analyzed by HPLC for the quantitative determination of acyclovir. The encapsulation efficiency and loading capacity of acyclovir-loaded chitosan NS were calculated using the equations below.

(1) $$\mathrm{Encapsulation}\;\mathrm{efficiency}\;\%=\frac{\mathrm{Amount}\;\mathrm{of}\;\mathrm{acyclovir}\;\mathrm{in}\;\mathrm{NS}}{\mathrm{Weight}\;\mathrm{of}\;\mathrm{acyclovir}}\times 100$$

(2) $$\mathrm{Loading}\;\mathrm{capacity}\;\%=\frac{\mathrm{Amount}\;\mathrm{of}\;\mathrm{acyclovir}}{\mathrm{Weight}\;\mathrm{of}\;\mathrm{acyclovir}-\mathrm{loaded}\;\mathrm{NS}}\times 100$$

### 2.8. Thermal Analysis

Differential Scanning Calorimetry (DSC) was carried out by means of a Perkin Elmer DSC/7 differential scanning calorimeter (Perkin-Elmer, Waltham, MA, USA) equipped with a TAC 7/DX instrument controller (Perkin-Elmer). The instrument was calibrated with indium for melting point and heat of fusion. A heating rate of 10 °C/min was employed in the 25–300 °C temperature range. The standard aluminum sample pans (Perkin-Elmer) were used. An empty pan was used as a reference standard. Analyses were performed in triplicate using about 3 mg freeze-dried samples under nitrogen purge.

### 2.9. In Vitro Drug Release Kinetics

The in vitro release kinetics studies were carried out using a multi-compartment rotating disk composed by several donor cells on one side separated from receiving cells by a hydrophilic dialysis membrane (Spectra/Pore, Spectrum^®^, cut-off 12,000–14,000 Da).

The donor phase consisted of 1 mL of acyclovir-loaded NS nanosuspension in PBS solution (pH 5.5) at a concentration of 5 mg/mL. The receiving phase consisted of PBS solution at pH 5.5. It was completely withdrawn at fixed times (15 min, 30 min, 45 min, 1 h, 2 h, 3 h, 4 h, and 6 h) and replaced with the same amount of fresh PBS solution. The concentration of acyclovir in the withdrawn samples was detected by HPLC.

The kinetic release behavior of acyclovir loaded in NS was compared to a free acyclovir phosphate buffer solution. The experiment was carried out in triplicate.

### 2.10. In Vitro Permeation Studies

The in vitro acyclovir permeation was studied using vertical static glass Franz cells purposely equipped with slices of ear pig skin to mimic the *stratum corneum* properties.

A hydrophilic gel consisting of 1% hydoxyethylcellulose was prepared and subsequently mixed 1:1 with acyclovir-loaded aqueous formulation. The drug permeation through ear pig skin from the acyclovir-loaded NS gel formulation and from a commercial cream formulation (Aciclovir Mylan Generics, Mylan S.p.A., Milano, Italy) were evaluated. Skin slices (1 mm thick) were isolated with a dermatome from the inner side of pig ears and then frozen at −18 °C. Before the experiments, the thawed skin was equilibrated in saline solution (NaCl 0.9% *w/w*), which had sodium azide (0.01%) added to preserve the skin at 25 °C for 30 min. After washing with saline solution, the skin layer was inserted between the two compartments of the vertical glass Franz cell with the *stratum corneum* side facing towards the donor chamber. The Franz cell was magnetically stirred at 1000 rpm and maintained at a constant temperature of 32 ± 1 °C using jacketed cells and a thermosetting system. The donor compartment contained 0.5 g of formulation.

The receptor phase consisted of a phosphate saline buffer 0.05 M (pH 5.5). At fixed times, the receptor phase (5 mL) was completely withdrawn and immediately replaced with an equal volume of fresh buffer.

The amount of acyclovir accumulated in the skin was quantitatively determined at the end of the in vitro experiment after skin slice extraction with methanol. Each experiment was repeated three times.

### 2.11. Biological Assays

#### 2.11.1. Cells

African green monkey kidney cells (Vero) (ATCC CCL-81) were purchased from the American Type Culture Collection (ATCC; Manassas, VA, USA). The culture medium for cells was Eagle’s minimal essential medium (MEM) (Gibco/BRL, Gaithersburg, MD, USA) supplemented with heat-inactivated 10% fetal calf serum (FCS) (Gibco/BRL) and 1% antibiotic-antimycotic solution (Zell Shield, Minerva Biolabs GmbH, Berlin, Germany).

#### 2.11.2. Viruses

The neurovirulent strains LV [20] and MS (American Type Culture Collection cat. no. VR-540) of HSV-1 and HSV-2, respectively, were used for in vitro experiments. Both strains were sensitive to acyclovir. Viral stocks were generated as previously described by Donalisio and collaborators [21]. Both strains were titrated by plaque assay on Vero cells.

#### 2.11.3. Cell Viability Assay

Vero cells were cultured in 96-well plates and incubated with acyclovir, acyclovir-loaded NS, and only NS at different concentrations and in triplicate. Cells were cultured under the same experimental conditions used for the in vitro antiviral assays and viability was determined by the CellTiter 96 Proliferation Assay Kit (Promega, Madison, WI, USA) according to the manufacturer’s instructions. Absorbances were measured using a Microplate Reader (Model 680, BIORAD, Hercules, CA, USA) at 490 nm. The effect on cell viability of NS at different concentrations was expressed as a percentage by comparing absorbances of treated cells with cells incubated with culture medium alone. The 50% cytotoxic concentrations (CC_50_s) and 95% confidence intervals (CIs) were determined using Prism software (Graph-Pad Software, San Diego, CA, USA).

#### 2.11.4. Cytotoxicity Assay

The cytotoxic effect of acyclovir, acyclovir-loaded NS, and only NS on Vero cells was measured by analyzing the release of lactate dehydrogenase (LDH) in the culture medium of treated cells. According to the manufacturer’s protocol of the LDH Cytotoxicity Detection Kit (TAKARA Bio Inc., Kusatsu, Japan), the lactate dehydrogenase release into the cell culture supernatant is directly proportional to the accumulation of dead cells [22].

#### 2.11.5. Virus Yield Reduction Assay

Vero cells were seeded in 24-well plates at a density of 10 × 10^4^ cells. After 24 h, cells were infected in duplicate with HSV-1 or HSV-2 at a multiplicity of infection (MOI) in 0.01 plaque-forming units (PFU)/cell. Following virus adsorption (2 h at 37 °C), the virus inoculum was removed and cells were washed and exposed to acyclovir or acyclovir-loaded NS until untreated cultures displayed extensive cytopathology. Supernatants were harvested and cell-free virus infectivity titers were determined in duplicate by the plaque assay in Vero cell monolayers. The end-points of the assay were the inhibitory concentrations that reduced the virus yield by 50% (IC_50_) versus the untreated virus control. The IC_50_ values for inhibition curves were calculated by using the program PRISM 4 (GraphPad Software, San Diego, CA, USA) to fit a variable slope-sigmoidal dose–response curve. A selectivity index (SI) was calculated by dividing the CC_50_ by the IC_50_ value.

#### 2.11.6. Evaluation of Cellular Uptake by the Confocal Laser Scanning Microscopy

Exponentially growing Vero cells were plated and cultured overnight in 24-well plates on glass coverslips. Subsequently, the cell monolayers were incubated with 100 µM of the labeled NS for the indicated time points and then extensively washed with PBS for the observation of the living cells. Confocal sections were taken on an inverted Zeiss LSM510 fluorescence microscope (Zeiss, Jena, Germany).

#### 2.11.7. Statistical Analysis

The results are expressed as mean ± SD. Statistical analyses were performed using an unpaired Student’s *t*-test. A value of *p* < 0.05 was considered significant.

## 3. Results and Discussion

In this research, chitosan nanospheres were designed as delivery systems for acyclovir topical administration. Chitosan-based nanocarriers have been widely studied for the unique properties of this polymer and, in particular, for its capability to interact with various epithelia and muco-adhesion properties. Several past studies referred to chitosan-based nanocarriers for skin and mucosal drug delivery [23,24,25].

Chitosan has been exploited for the delivery of acyclovir by Hasanovic et al. who developed chitosan-tripolyphosphate nanoparticles to enhance the chemical stability of the drug [24]. Chitosan nanospheres with enhanced characteristic were prepared by combining a previous tuned method based on ionic gelation [19] and the nano-emulsion templating method [26]. This peculiar experimental set-up permitted to obtain stable chitosan nanospheres is able to load acyclovir to a great extent. As already reported, this innovative preparation procedure has been tailored to guarantee high reproducibility in the formulation process, which reduces manual mistakes.

The average diameter, polydispersity index, zeta potential, encapsulation efficiency, and loading capacity values of the three types of NS formulations (loaded, blank, and fluorescent) are reported in Table 1.

The drug loading of acyclovir was found to be (8.5%) with an encapsulation efficiency of about 87%. This result further confirmed the success of the proposed experimental set up and allowed an enhancement of the drug loading amount in comparison to previous investigations [24].

DSC analysis showed that the drug is molecularly dispersed in the polymer matrix due to the complete disappearance of the melting endotherm peak at 255 °C of acyclovir, which suggests a complete amorphization of the drug in the carrier (Data not shown).

**Figure 1:**
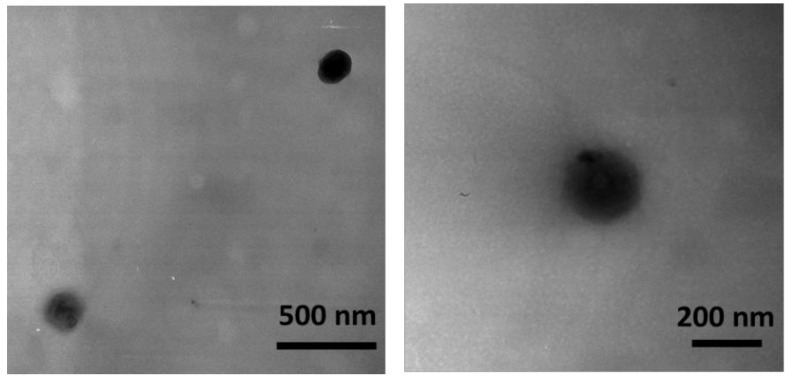
Transmission electron microscopy (TEM) image of acyclovir-loaded chitosan nanoparticles.

The TEM micrograph of the acyclovir loaded NS is reported in Figure 1. It shows that nanoparticles have a spherical shape and nearly smooth surface structures. TEM analysis also revealed that acyclovir-loaded chitosan NS are discrete and non-aggregated.

**Figure 2:**
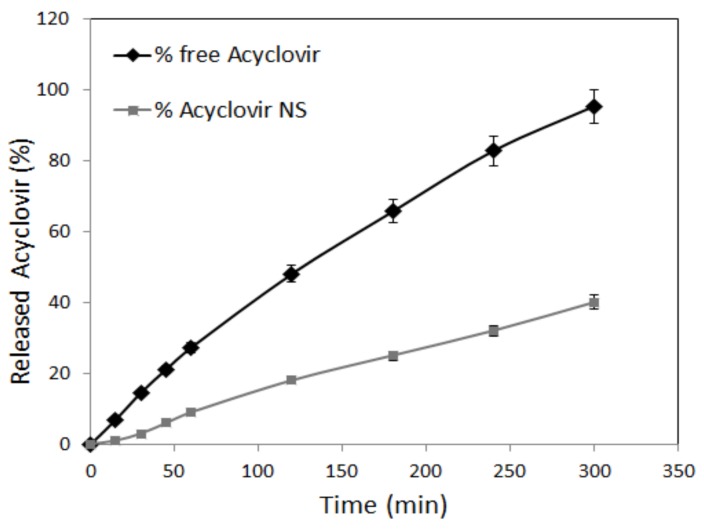
In vitro release kinetics of acyclovir from the drug aqueous solution and from the chitosan acyclovir NS.

The In vitro release study (Figure 2) showed a prolonged release kinetics of acyclovir-loaded NS compared to the free acyclovir aqueous solution. After 3 h, about 20% of acyclovir was released from chitosan NS.

**Figure 3:**
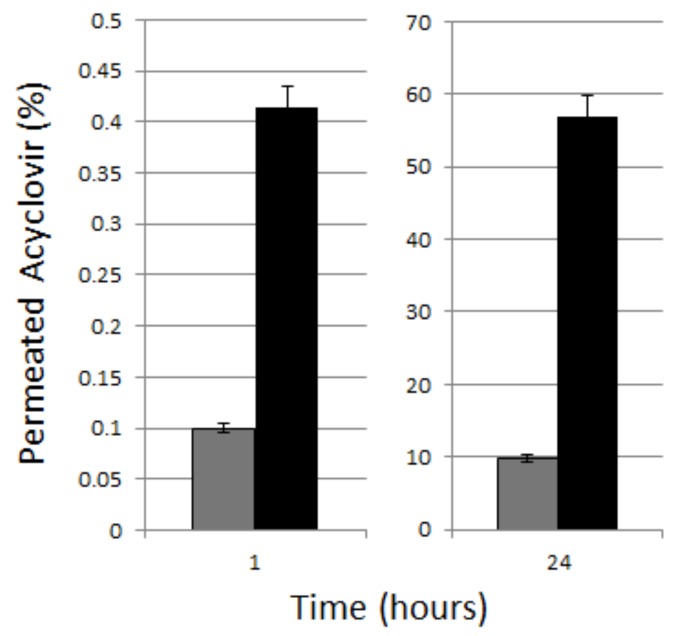
Comparison of Acyclovir permeation through porcine skin from the chitosan NS gel and commercial cream.

The in-vitro permeation studies were performed comparing the acyclovir-loaded NS gel formulation and a commercial cream formulation for 24 h. Figure 3 displays the percentage of the permeated acyclovir through the porcine skin after 1 h and after 24 h of administering the semisolid formulations. The results confirmed the enhancement of acyclovir permeation through the skin when delivered by the chitosan NS gel. In fact, after 1 h, a significant difference between the amount of acyclovir permeated after the release from the commercial cream and from the chitosan NS gel was observed. In the former case, about 0.1% of the drug permeated through the porcine skin while in the latter this value was up to 0.4%. The same trend was confirmed after 24 h when the acyclovir released from the chitosan NS gel was at about 55%, which is remarkably higher than the 10% that permeated from the commercial cream. The amount of acyclovir accumulated in the skin was quantitatively determined at the end of the in vitro experiment after the skin slice extraction method using methanol. The quantitative analysis showed that the acyclovir accumulation in the pig skin was about 1% for the drug commercial formulation and about 30% for the acyclovir released from the chitosan NS gel after 24 h.

**Figure 4:**
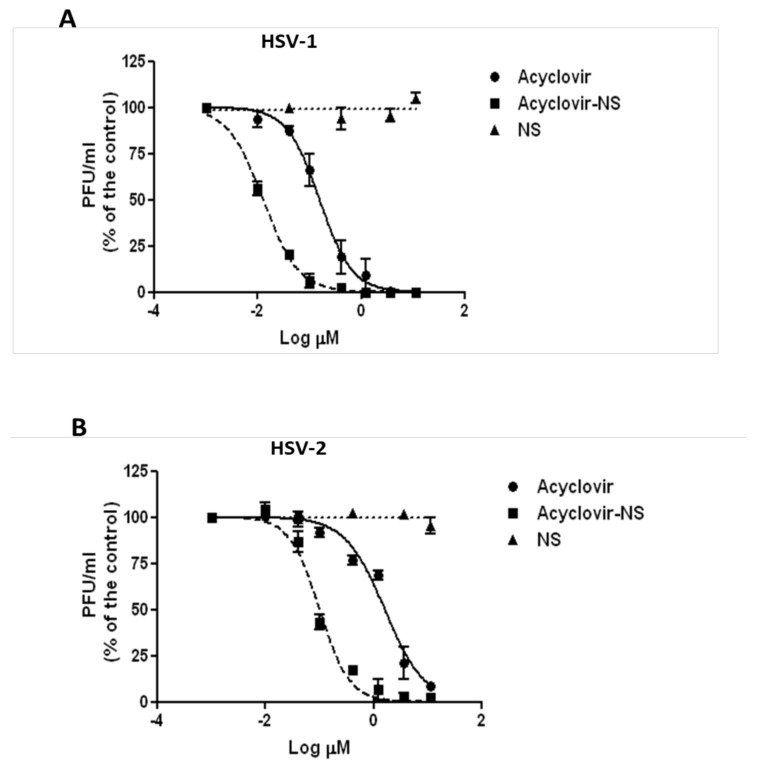
Antiviral activity of free acyclovir, acyclovir-loaded NS, and plain carrier (NS) against HSV-1 (**A**) and HSV-2 (**B**). Vero cells were infected at a multiplicity of infection (MOI) of 0.01 and then exposed to different drug concentrations. Virus titers in the supernatants of cell cultures were determined by a standard plaque assay at 48 h or 24 h post-infection for HSV-1 or HSV-2, respectively. Values are the means of three separate determinations.

As far the biological results are concerned, virus yield reduction assays were performed to compare the antiviral activity of plain acyclovir and acyclovir-loaded NS against HSV-1 and HSV-2 infections. Both viruses were sensitive to acyclovir. This antiviral assay evaluates the production of infectious viruses after multiple cycles of viral replication. The assay was performed on monolayers of Vero cells incubating serial dilutions of tested formulation after viral adsorption. As shown by the dose-response curves in Figure 4, the acyclovir-NS complex displayed higher antiviral activity than the activity of free acyclovir against both HSV-1 and HSV-2 infections. In particular, IC_50_ values against HSV-1 determined at 48 h post infection were found to be 0.012 µM (95% CI: 0.011–0.014) for acyclovir-loaded NS and 0.156 µM (95% CI: 0.123–0.197 µM) for free acyclovir while those obtained against HSV-2 determined at 24 h post-infection were 0.100 µM (95% CI: 0.081–0.124 µM) for acyclovir-loaded NS and 1.608 µM (95% CI: 1.255–2.060 µM) for free acyclovir. Virus titers were determined at 48 h or 24 h post-infection for HSV-1 or HSV-2 respectively due to the different replication time of the two viruses. The IC_50_ values for plain acyclovir are consistent with previously published values [27,28]. No antiviral activity was exerted by the unloaded NS at tested doses (see Figure 4).

**Figure 5:**
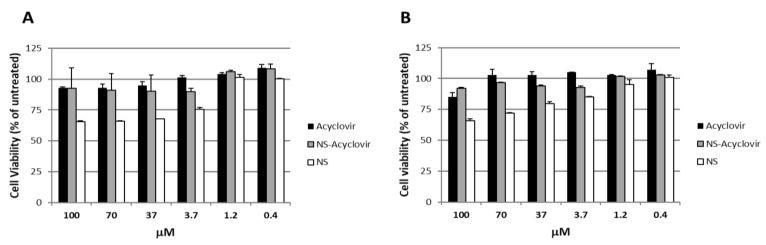
Effect of acyclovir, acyclovir-loaded NS, and only NS on the viability of non-infected Vero cells as a function of the drug concentration at 24 h (**A**) and 48 h (**B**). *X* axis: NS concentration, *Y* axis: cell viability (% of untreated control). Each point represents the mean ± SD (*n* = 3).

To exclude that the inhibitory activities observed were due to cellular alterations, a cell viability assay and a cell cytotoxicity assay were performed on treated Vero cells under the same experimental conditions used for antiviral assays. As reported in Figure 5, acyclovir-loaded NS did not affect cell viability at the concentrations and time points chosen. On the other hand, the plain carrier shows a weak inhibition of cellular proliferation at high doses after 24 h and 48 h post treatment.

**Figure 6:**
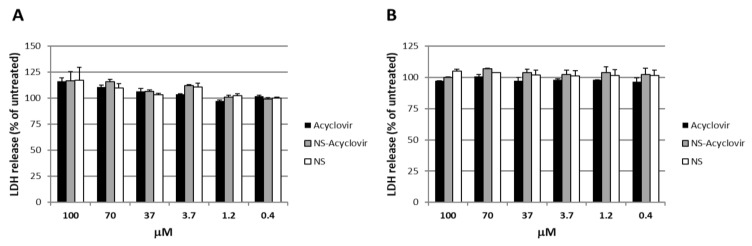
Cytotoxic effect of acyclovir, acyclovir-loaded NS, and NS alone on non-infected Vero cells by measuring the release of lactate dehydrogenase in culture medium at 24 h (**A**) and 48 h (**B**). *X* axis: nanospheres concentration, *Y* axis: release of lactate dehydrogenase (% of untreated control). Each point represents the mean ± SD (*n* = 3).

The cell cytotoxicity assay was carried out by measuring the level of LDH. A stable cytoplasmic enzyme that is released into the cell culture supernatant upon damage of the cytoplasmic membrane. No significant difference in the release of the cytoplasmic enzyme LDH was observed between formulations-treated cells and untreated cells, which indicates no signs of cytotoxicity induced by NS (see Figure 6).

These experiments revealed that the acyclovir-loaded NS are not cytotoxic with CC_50_ values (50% cytotoxic concentration) higher than 100 µM and the selectivity index (SI) greater than 7936 or 1000 for HSV-1 and HSV-2, respectively.

**Figure 7:**
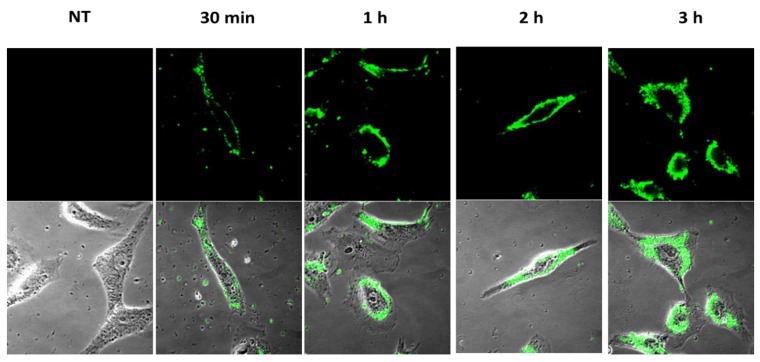
Cell uptake of fluorescent NS. Vero cells were incubated with the formulation for the times indicated and then analyzed by confocal laser scanning microscopy without fixation. The upper panels show the fluorescence images while the lower panels show fluorescence images merged with phase-contrast images. The first column on the left shows the control cells, which were not incubated with the formulation (NT = no treated).

In order to investigate whether NS could penetrate inside cells and deliver acyclovir, we examined Vero cells treated with fluorescent nanoparticles by using a confocal laser scanning microscopy. The assay was carried out on living unfixed cells to avoid misleading due to the cell fixation protocols. As shown in Figure 7, virtually all NS were internalized in the cells and localized in the cytoplasm within 3 h from treatment with a preferential accumulation in the perinuclear compartment. Other studies on particulate delivery systems described a perinuclear accumulation of nanoparticles after internalization in cells [29,30,31]. Fluorescence was not detected in control cells that had not been exposed to the labeled compounds.

## 4. Conclusions

The present work aimed at developing a successful new topical formulation for the delivery of acyclovir was able to increase the efficacy of the currently available commercial products for the topical treatment of Herpes simplex virus infections. For this purpose, chitosan, which is a natural linear polycationic polysaccharide, was selected due to its biocompatibility, low toxicity, and biodegradability. Moreover, chitosan is an interesting biomaterial for dermatological applications since it can play a key role in wound healing and regeneration processes. In this research study, a purposely modified nano-emulsion templating method was used as an innovative experimental set up to prepare chitosan nanospheres loaded with acyclovir. In comparison with previous scientific investigations, the proposed chitosan nanoparticles system showed improved characteristics such as enhanced drug loading amount, prolonged release kinetics, no cytotoxicity, and an improved in vitro antiviral efficacy. Moreover, the chitosan nanospheres tuned in this work were successfully able to act as skin surface reservoir of acyclovir, which enhance its skin permeation in comparison with a commercial topical formulation. This mechanism of action allows an improved topical delivery of anti-infectives, which are delivered to the stratum corneum or epidermis and retained there for a prolonged time against microbials. Furthermore, in vitro biological studies demonstrated a higher antiviral activity of the acyclovir-loaded chitosan nanospheres than that of free acyclovir against both the HSV-1 and HSV-2 strains. These promising results underline the possibility to increase the efficacy of the currently available acyclovir topical formulations using this new nano-technological approach.

## Figures and Tables

**Table 1:** Summary of the physico-chemical characterization of Acyclovir-loaded chitosan nanospheres (NS), blank NS, and fluorescent NS. PDI: Polydispersity Index; ZP: Zeta Potential.

Parameters	Acyclovir NS	Blank NS	Fluorescent NS
Average diameter ± SD (nm)	200.0 ± 5.0	205.0 ± 5.2	285.0 ± 4.9
Polydispersity Index(PDI)	0.10	0.10	0.12
Zeta Potential (ZP) ± SD (mV)	40.6 ± 10.6	38.6 ± 16.5	38.6 ± 16.5
Encapsulation efficiency (%)	86 ± 1.45		
Loading capacity (%)	8.5		
